# Complementarity of BOLD and ADC‐fMRI in Mapping Brain Visual Processing in the Rat

**DOI:** 10.1002/nbm.70231

**Published:** 2026-02-03

**Authors:** Jean‐Baptiste Pérot, Andreea Hertanu, Arthur Spencer, Jasmine Nguyen‐Duc, Nikolaos Molochidis, Valerio Zerbi, Maxime Yon, Ileana Jelescu

**Affiliations:** ^1^ Department of Radiology Lausanne University Hospital (CHUV) Lausanne Switzerland; ^2^ Faculty of Biology and Medicine University of Lausanne (UNIL) Lausanne Switzerland; ^3^ Department of Psychiatry, Faculty of Medicine University of Geneva Geneva Switzerland; ^4^ Department of Basic Neurosciences, Faculty of Medicine University of Geneva Geneva Switzerland; ^5^ Université de Rennes Rennes France

**Keywords:** diffusion, fMRI, visual system

## Abstract

The transition from static to dynamic vision is encoded in the superior colliculus (SC), as recently shown using blood oxygen level–dependent functional magnetic resonance imaging (BOLD‐fMRI) of the rat brain. Visual stimulation at a higher frequency than the flicker fusion frequency threshold is associated with a negative BOLD response in the visual cortex, triggered by the SC. Here, we explored this mechanism in further depth using visual stimulation at low (1 Hz) and high (25 Hz) frequencies in the rat. We used both BOLD‐fMRI and apparent diffusion coefficient (ADC)–fMRI to yield complementary information on brain activity during visual stimulation, from neurovascular and neuromorphological coupling perspectives. We compared responses between different brain regions (the dorsolateral geniculate nucleus, the medial and lateral parts of the SC, the corpus callosum, and the visual cortex), sexes, and field strengths (9.4 and 14 T). Results confirmed distinct BOLD responses to low‐ and high‐frequency stimulation and highlighted for the first time that the transition from static to dynamic vision is characterized by negative BOLD in the lateral SC specifically. Whereas the BOLD response to visual stimulation depends on the vasculature properties across brain regions and sexes, we found a significant ADC‐fMRI response (in the form of reduced ADC during excitatory activity) in the SC that was more consistent across visual frequencies, as well as in the corpus callosum, to which BOLD was not sensitive. Our results support an interplay between neural activity and hemodynamic response underlying the transition from static to dynamic vision, best characterized using two fMRI contrasts.

## Graphical abstract

BOLD‐fMRI and ADC‐fMRI were acquired at 9.4 and 14 T during visual stimulation with variable frequency.

BOLD‐fMRI showed robust positive/negative responses but depended on regional vascularization patterns. ADC‐fMRI was able to detect neural activity independently from vascular effects at 9.4 T.

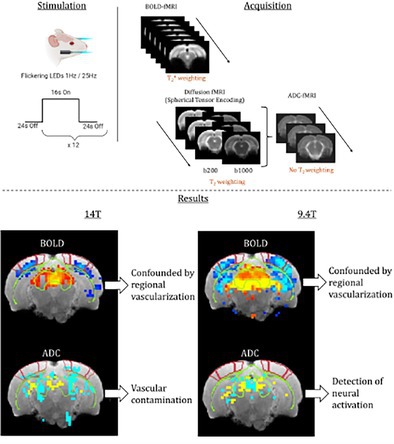

## 1 Introduction

The visual system of the mammalian brain exhibits remarkable plasticity, enabling adaptive responses to various environments and stimuli. The decoding and extraction of visual features for high‐order tasks are established by the cross‐talks between regions of subcortical and cortical gray matter constituting the visual pathway [1, 2]. Retinal neurons project mainly to the dorsolateral geniculate nucleus (DLGn) of the thalamus and to the superior colliculus (SC), which are both interconnected with the visual cortex [3, 4]. Although the functional architecture of these regions has been described [5], it is still unclear how their interplay drives the adaptive response to varying stimuli. In particular, the mechanisms underlying the transition from perception of individual events to a dynamic vision, for instance, of moving objects, are still under investigation. This transition can be described by the flicker fusion frequency (FFF) threshold, which is defined for a flashing stimulus as the frequency at which the individual stimuli are perceived as one [6, 7].

Using blood oxygenation level–dependent (BOLD) functional magnetic resonance imaging (fMRI) in rats, Gil et al. (2024) recently showed that this transition is encoded in the SC [8]. Indeed, this was the only brain region that switched from a positive to a negative BOLD response with increasing frequency of the visual stimulus—the transition occurring around 18 Hz. However, the BOLD‐fMRI contrast relies on neurovascular coupling and is the result of an interplay between changes in local blood flow, volume, and oxygenation, aimed at supporting the metabolic demands of firing neurons [9, 10, 11]. Four mechanisms contribute to the BOLD signal, two of which are intravascular and two are extravascular [12]. For either of the compartments, static dephasing and dynamic dephasing contribute to GRE‐BOLD signal, whereas only the dynamic component contributes to SE‐BOLD. Furthermore, the association of positive and negative BOLD responses with excitatory and inhibitory brain activity, respectively, is still under investigation [13, 14, 15]. Finally, the sensitivity of BOLD‐fMRI is reduced in less vascularized areas such as white matter [16, 17].

Apparent diffusion coefficient (ADC)–fMRI [18] has been proposed as an alternative contrast to probe neuronal activity more directly than its BOLD counterpart by recording changes in the diffusion properties of the tissue resulting from neuromorphological coupling, i.e., microstructure fluctuations concomitant with neuronal activity [19, 20, 21]. However, achieving sensitivity to these microstructure fluctuations without vascular effect remains challenging. In particular, the microcirculation phenomenon, as commonly measured in intravoxel incoherent motion (IVIM) imaging, is a potential bias for ADC calculation [22, 23]. Recent advances in the acquisition schemes have allowed the minimization of spurious hemodynamic contributions to the ADC‐fMRI time courses [24, 25]. In addition to minimizing vascular confounds in gray matter, ADC‐fMRI has recently demonstrated the ability to detect neuronal activation in the human brain in response to motor and visual block stimulations with enhanced sensitivity to white matter, as compared to BOLD [25, 26]. In addition, computing ADC based on high (e.g., *b* = 1000 s/mm^2^) and nonzero (e.g., *b* = 200 s/mm^2^) *b*‐values limits the sensitivity to IVIM effect

In the present work, we used a visual block‐stimulation paradigm in the rat at two different frequencies: a low frequency of 1 Hz at which the visual pathway operates in static vision mode (every flash is encoded as a separate event) and a high frequency of 25 Hz reported to induce a shift to dynamic vision mode (flashing stimuli are fused together) [8]. Implementing the latest vascular mitigation strategies for ADC‐fMRI, we aimed to assess potential complementarity between BOLD‐ and ADC‐fMRI contrasts in mapping region‐specific brain activity changes associated with FFF, from a vascular and neuronal perspective. This paradigm is ideal as it presents a shift from positive to negative BOLD responses in the SC, for which the ADC‐fMRI signature is unknown. Furthermore, we investigated the dependencies of the activation maps on sex (males vs. females) and on magnetic field strength.

## 2 Materials and Methods

### 2.1 Animal Experiments

#### 2.1.1 Animals

All experiments were conducted in respect of the Swiss law for protection of animals (OPAn) and were authorized by cantonal (authorization n° VD3958B) and national (authorization n° 36,256) ethics committees.

Sprague–Dawley rats (*n* = 18, 12 females and 6 males, 200–225 g, 8–10 weeks old) were received from Charles River Laboratories. Rats were housed in cages of 2 (males) or 3 (females) with a 12‐h day/night cycle, water and food ad libitum, and nesting enrichment. All experiments were performed after a minimum of 1 week of acclimatization. Animals were split into three groups. The first and second groups consisted of six females and six males, respectively, and were scanned on a 14 T Bruker MRI system. The third group, constituted of six females, was scanned on a 9.4 T Bruker MRI system. The number of six animals per group was decided based on other studies with similar sample size [27]. No exclusion or inclusion criterion was used; data from all animals were included.

#### 2.1.2 Anesthesia and Monitoring Protocol

Rats were initially anesthetized with isoflurane at 4% in a 70/30% air–oxygen mixture. Isoflurane was reduced to 2% after induction. Rats were then transferred and fixed using a bite bar and ear bars to a homemade MRI cradle. Eye ointment was applied. Rats were shaved on the back and a catheter (22G) was inserted subcutaneously for medetomidine administration. Body temperature was continuously monitored using a rectal thermometer and maintained around 37°C ± 0.5°C using a warm water circulation system. Respiratory rate was also continuously monitored using a respiration pillow placed under the animal's thorax and connected to a monitoring system. After installation of the rats in the MRI system, the anesthesia was switched from isoflurane to medetomidine. First, a bolus of medetomidine (0.1 mg/kg) was injected via the subcutaneous catheter connected to a perfusion line, and isoflurane was reduced to 0% gradually during the following 10 min [28, 29, 30] aiming for a respiration rate ~80–85 bpm. Fifteen minutes after bolus administration, a continuous subcutaneous infusion of medetomidine (0.1 mg·kg^−1^·h^−1^) was started and maintained until the end of the MRI experiment [31]. Maximum duration of the medetomidine anesthesia was 3 h. At the end of the last scan, the medetomidine perfusion was switched off and an intramuscular injection of atipamezole (0.5 mg/kg) was performed as antagonist to medetomidine before returning the rat to its cage.

### 2.2 Visual Stimulation

Visual stimulation setup was similar for both 9.4 T and 14 T MRI scanners. A bifurcated optic fiber connected to a blue LED (λ = 470 nm and *I* = 8.1 × 10–1 W/m^2^, Doric Lenses, Quebec, Canada) was placed horizontally in front of each eye of the animal in the MRI bed for binocular visual stimulation during the MRI experiment. The LED was piloted by an Arduino connected to the MRI output trigger. fMRI sequences triggered the following visual block‐stimulation paradigm: after an initial 24 s of rest, epochs of 16 s of bilateral flashing light and 24 s of rest were repeated 12 times for a total duration of 8 min 24 s. The Arduino was resynchronized with the MRI trigger after each epoch to avoid accumulation of delay between the MRI and the internal Arduino clock. Visual stimulus was flashing at a frequency of either 1 or 25 Hz, with a flash duration of 10 ms [8].

### 2.3 MRI Experiments

#### 2.3.1 14 T MRI

**FIGURE 1:**
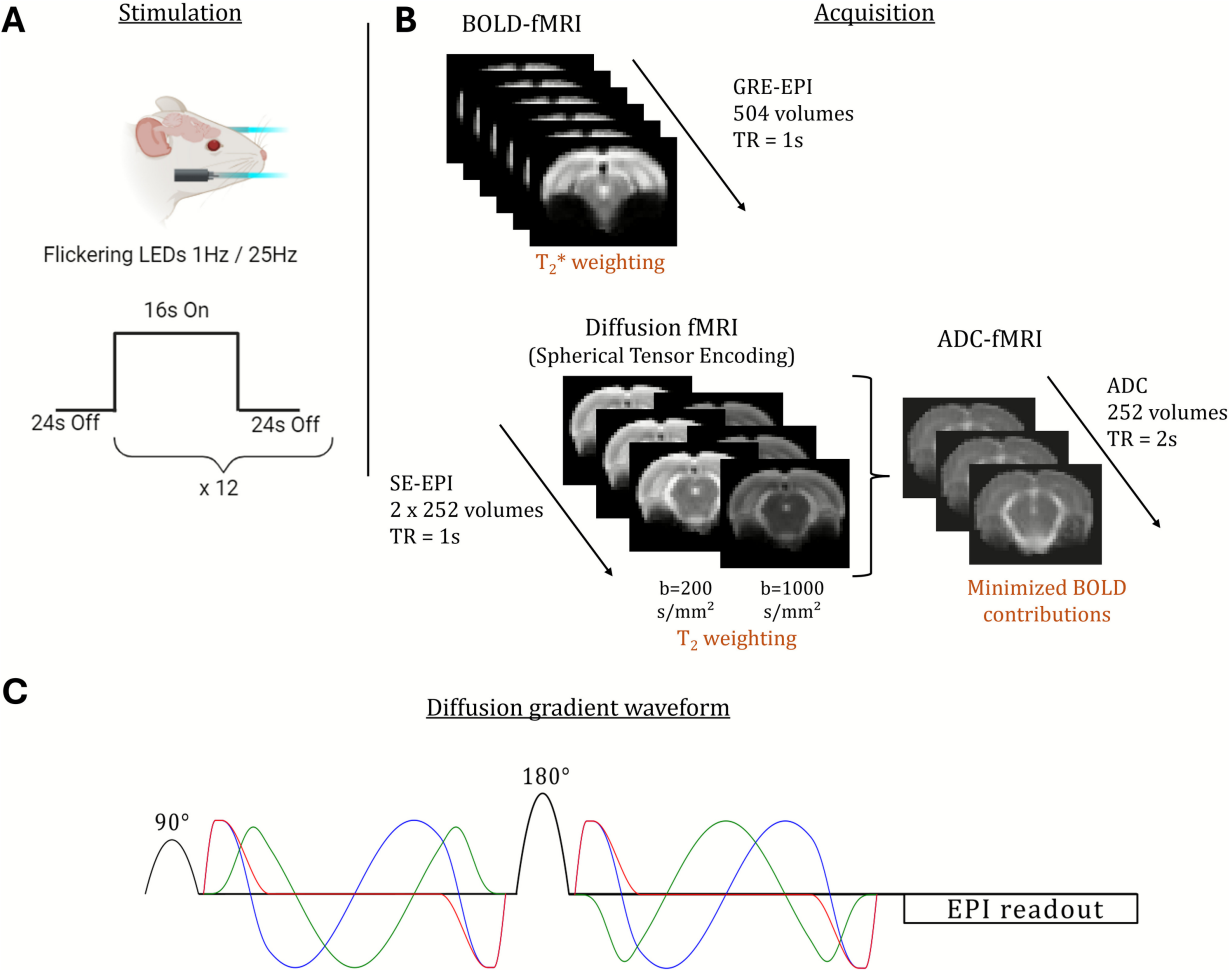
Design of the study. (A) Block‐stimulation paradigm. (B) Functional MRI acquisition design for BOLD‐ and ADC‐fMRI. (C) Sequence diagram of the dw‐SE‐EPI sequence used for ADC‐fMRI. Minimized BOLD contributions are achieved by estimating the ADC at each time point from two diffusion‐weighted images, acquired with *b*‐values above the IVIM regime and using diffusion gradient waveforms compensated for cross‐terms with background susceptibility gradients.

The first and second groups were scanned on a 14 T Bruker MRI system (*G*_max_ = 1000 mT/m, *S*_max_ = 5600 T·m^−1^·s^−1^) operating on ParaVision 360 v1.1 software (Bruker, Ettlingen, Germany) and using a volume coil for transmission and a two‐channel surface coil for reception (Rapid Biomedical GmbH, Rimpar, Germany). After animal installation and switch of anesthesia, a *B*_0_ field map was acquired and used for shimming in an ellipsoidal volume covering the rat brain using MapShim. A T_2_‐weighted (T_2_‐w) image was acquired for anatomical reference (TurboRARE, TR/TE = 2500/6 ms, resolution 0.125 × 0.125 × 0.5 mm^3^, matrix 160 × 160, 45 slices, duration = 6 min 40 s). fMRI acquisitions started at least 30 min after the switch of anesthesia from isoflurane to medetomidine to allow for isoflurane clearance. BOLD‐fMRI was acquired using a gradient echo planar imaging sequence (GRE‐EPI, TR/TE = 1000/11 ms, resolution 0.38 × 0.38 × 1.5 mm^3^, matrix 68 × 68, 10 slices, 504 repetitions) and ADC‐fMRI using a diffusion‐weighted spin echo EPI sequence with two alternating *b*‐values (dw‐SE‐EPI, TR/TE = 1000/41 ms, matched geometry with GRE‐EPI, *b*_1_ = 200 s/mm^2^, *b*_2_ = 1000 s/mm^2^, 504 repetitions). To avoid fiber‐orientation bias, spherical tensor encoded diffusion weighting was used to achieve isotropic diffusion encoding in each measurement [26, 32]. Thus, no repetition was needed with different directions in this experiment, as every measurement was weighted in all directions equally, thanks to the use of spherical encoding gradient waveforms. The gradient waveforms were also cross‐term compensated to minimize contributions from background blood susceptibility gradients [33]. Figure 1 presents a summary of our block‐stimulation paradigm, acquisition scheme, and a sequence diagram showing the shape of the gradient waveform used to achieve spherical encoding. Eight fMRI runs were acquired in total for each rat: BOLD‐fMRI and ADC‐fMRI sequences were acquired twice for each stimulation frequency, in a randomized order. Twelve dummy repetitions were acquired and discarded in the beginning of every scan to reach steady state. Forward and reverse phase encoded images were also acquired for GRE‐EPI and for dw‐SE‐EPI with a null *b*‐value (*b*_0_ image) to enable EPI spatial distortion correction during preprocessing.

#### 2.3.2 9.4 T MRI

The third group (*n* = 6 females) was scanned on a 9.4 T Bruker MRI system (*G*_max_ = 660 mT/m, *S*_max_ = 4570 T·m^−1^·s^−1^) operating on Paravision 360 v3.5 software, using a volume coil for transmission and a six‐channel surface cryoprobe for reception. After installation, the shimming procedure was identical to that on the 14 T system. Acquisition parameters for the anatomical image, GRE‐EPI, and dw‐SE‐EPI were kept as consistent as possible to the 14 T protocol. Some variations were necessary to accommodate hardware differences between the two systems. Anatomical reference (TurboRARE, resolution 0.14 × 0.14 × 1.1 mm^3^, 256 × 256, 20 slices, duration = 10 m 40 s), GRE‐EPI (TR/TE = 1000/10 ms, resolution 0.36 × 0.36 × 1.5, matrix 69 × 50 with saturation band, eight slices), and dw‐SE‐EPI (TR/TE = 1000/42 ms, matched geometry with GRE‐EPI) were acquired using the same protocol. The same gradient waveforms as previously were used for diffusion‐weighting with amplitude rescaling to adapt to the 9.4 T gradient limitations. Table 1 presents a summary of sequence parameters used with both 9.4 and 14 T setups.

**TABLE 1:** Sequence parameters used for anatomical reference and functional MRI, at 9.4 and 14 T.

	Anatomical reference		BOLD‐fMRI		ADC‐fMRI	
	9.4 T	14 T	9.4 T	14 T	9.4 T	14 T
Sequence	TurboRARE		GRE‐EPI		dw‐SE‐EPI	
TR/TE (ms)	2500/8	2500/6	1000/10	1000/11	1000/42	1000/41
Resolution (mm^3^)	0.14 × 0.14 × 1.1	0.125 × 0.125 × 0.5	0.36 × 0.36 × 1.5	0.38 × 0.38 × 1.5	0.36 × 0.36 × 1.5	0.38 × 0.38 × 1.5
Slices	20	45	8		8	
*b*‐Values (s/mm^2^)	—		—		200, 1000	
Acquisition time (s)	640	400	504		504	

### 2.4 Image Preprocessing

The main steps of the following preprocessing pipeline are summarized in Supplementary Figure S1. Raw data extracted from Bruker Paravision software were converted to NIfTI using the Dicomifier toolbox (https://github.com/lamyj/dicomifier). Following this step, dw‐SE‐EPI time series were split per *b*‐values, leading to two different time series (b200 and b1000) of 252 repetitions with 2‐s temporal resolution each. Images from both b200 and b1000 time series were isotropically sensitized to diffusion. BOLD time series corresponded to GRE‐EPI time series of 504 repetitions with 1‐s temporal resolution. BOLD, b200, and b1000 time series underwent the same preprocessing steps including MP‐PCA denoising [34], Gibbs unringing [35], distortion correction [36], and motion correction (rigid + affine) [37].

Following these steps, the preprocessed b1000 time series was registered to the b200 time series, and the resulting aligned datasets were used to compute the ADC time series, as $\mathrm{ADC}\left(t\right)=\frac{1}{200-1000}\ln\left(\frac{S_{1000}\left(t\right)}{S_{200}\left(t\right)}\right)$. The brain was extracted from the T_2_‐w image using RATS [38] to yield a brain mask. The T_2_‐w image was then registered to functional images (average of all BOLD volumes for GRE‐EPI, average of all b200 repetitions for dw‐SE‐EPI) using ANTs [37], and the transformation was applied to the brain mask for skull stripping of functional time series. High‐pass filtering (100‐s cutoff) was applied to functional time series at this step. No spatial smoothing was performed.

Multivariate templates were generated from the T_2_‐w, *b*_0_, and average BOLD images of the six rats from the second cohort [39]. Individual functional time series were registered to their corresponding (*b*_0_ or BOLD) template using ANTs [37]. Waxholm Space Atlas of the rat brain was first registered to the T_2_‐w template and then propagated into subject space by applying the inverse transformations previously estimated in order to obtain for segmentation of the optic tract (Ot), corpus callosum (CC), DLGn, SC, primary and secondary visual cortices (V1 and V2), and retrosplenial cortex (RSC). The SC was manually segmented on the Waxholm Space Atlas between its medial and lateral parts using ITK‐snap (Supplementary Figure S2).

### 2.5 fMRI Analysis

#### 2.5.1 BOLD Activation Maps

Processed BOLD time series underwent general linear model (GLM) analysis at the subject level using a boxcar response function and cluster correction with threshold of |*Z*| > 2.3 and *p* < 0.05. As the hemodynamic response in the rat brain is much faster than in humans, the boxcar response provides a better fit than traditional HRF [40]. Results from the subject‐level GLM were then registered to the template space. A group‐level GLM analysis was performed separately per field strength, stimulation frequency, and, in the case of 14 T data, per sex on the registered *z* score maps, using a cluster‐correction of either |*Z*| > 1.5 or |*Z*| > 2.3 and *p* value threshold of 0.05. The same GLM analysis was also performed on dw‐SE‐EPI time series with the *b*‐value of 200 s/mm^2^ for comparison of activation maps obtained with GRE‐EPI and dw‐SE‐EPI.

In order to make results more directly comparable to ADC‐fMRI analysis (see below), BOLD time courses from female rats at 9.4 T also underwent GLM analysis using the finite impulse response (FIR) method.

#### 2.5.2 ADC Activation Maps

To avoid assumptions on the ADC‐fMRI response function, subject‐level GLM analysis was performed on ADC time series with boxcar response function convolved with FIR (four impulses, 16‐s window) [41] and cluster‐correction with threshold of |*Z*| > 2.3. As FIR analysis does not yield positive or negative contrasts like boxcar, responses in significant voxels were pooled across rats for each field strength and each stimulation frequency and classified using time‐series K‐means clustering with 10 clusters and k‐means++ initialization. The average response was extracted per cluster. Clusters with average positive or negative values during visual stimulation were pooled together for better visualization of voxels with a positive or negative ADC response.

#### 2.5.3 Average Response Plots

To visualize the average response to visual stimulation in specific brain regions, for each time series the signal was first averaged across voxels included in the mask of the region, normalized to the mean value of the first six volumes of each epoch, and averaged across epochs. Finally, the mean value and standard deviation across time series were computed and visually represented.

For better visualization and comparison with BOLD, ADC time series with a temporal resolution of 1 s were also generated. To that end, we first linearly interpolated the b200 and b1000 time series to a 1‐s resolution. A new ADC time series was then computed from pairs of one interpolated and one measured *b*‐value corresponding to the same time point in the series. The resulted interpolated ADC time series were used only for plotting the average ADC response. The GLM and FIR analyses described above were performed exclusively with the original 2‐s temporal resolution.

## 3 Results

### 3.1 BOLD‐fMRI Response to 1‐ and 25‐Hz Visual Stimuli Depends on Sex and Field Strength

**FIGURE 2:**
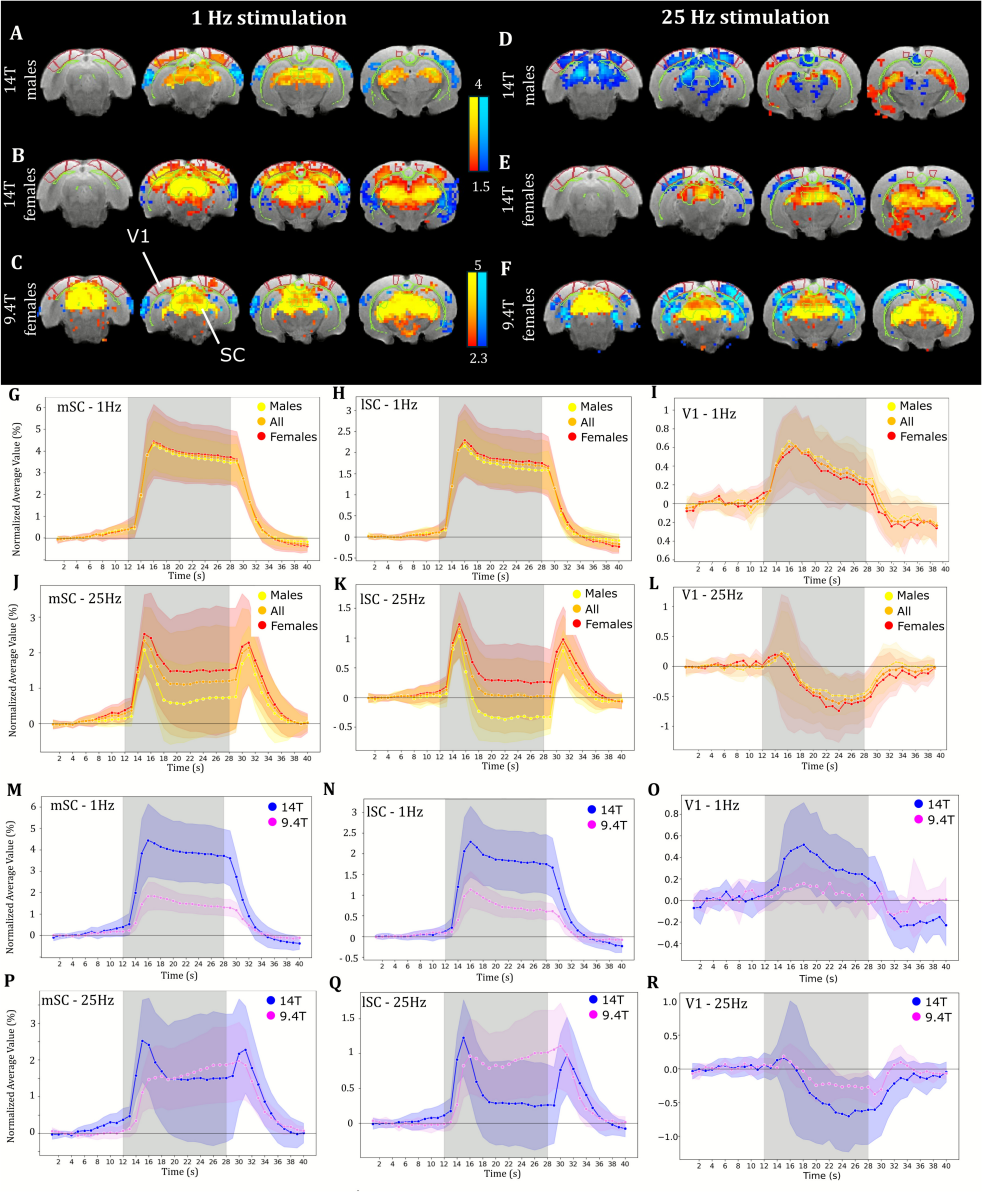
BOLD response to 1‐ and 25‐Hz visual stimulation in the brain of male and female rats at 14 and 9.4 T. (A–F) Cluster‐corrected group‐level activation maps of significant positive (red–yellow) and negative (blue–light blue) BOLD response to 1‐Hz (A–C) and 25‐Hz (D–F) visual stimulation in (A and D) males acquired at 14 T (*n* = 6, |Z| > 1.5, |*Z*|_max_ = 5.4), (B and E) females acquired at 14 T (*n* = 6, |*Z*| > 1.5, |*Z*|_max_ = 6.8), (C and F) females acquired at 9.4 T (*n* = 6, |*Z*| > 1.5, |*Z*|_max_ = 18.1). (G–I) Average response to 1‐Hz visual stimulation in (G) the medial SC, (H) the lateral SC, and (I) V1 in females (red, *n* = 6), males (yellow, *n* = 6), and all animals (orange, *n* = 12). (J–L) Average response to 25‐Hz visual stimulation in (J) the medial SC, (K) the lateral SC, and (L) V1. (M–O) Average response to 1‐Hz visual stimulation in (M) the medial SC, (N) the lateral SC, and (O) V1 in females acquired at 14 T (blue, *n* = 6) and 9.4 T (violet, *n* = 6). (P–R) Average response to 25‐Hz visual stimulation in (P) the medial SC, (Q) the lateral SC, and (R) V1. Shading denotes the standard deviation. Gray area represents stimulus duration.

Activation of the visual system was probed in male and female rats at 9.4 and 14 T using visual stimulation. Two different stimulation frequencies were used to investigate responses to static (1 Hz) and dynamic (25 Hz) visual stimuli. Visual stimulation with a frequency of 1 Hz (Figure 2A–C) induced a large area of strong positive BOLD response centered on the DLGn and SC, as well as a positive BOLD response with lower intensity in V1 and V2. Activation maps of females (Figure 2A) and males (Figure 2B) were spatially consistent; however, significant BOLD response covered a larger area in females than in males. Cortical areas with significant negative BOLD response were also found in a location corresponding to AuC. Activation maps were very similar in females acquired at 9.4 T (Figure 2C), although fewer significant cortical areas were found.

In response to 1‐Hz visual stimulation, the average BOLD response in the SC and V1 showed very similar temporal dynamics across sexes (Figure 2G–I) and field strengths (Figure 2M–O), differing only in response magnitude.

In response to 25‐Hz visual stimulation, the visual cortex presented a negative BOLD response, contrasting with 1‐Hz stimulation. This observation was spatially consistent in males (Figure 2D) and females (Figure 2E). In contrast, in the SC, activation maps showed a negative response in males and a positive response in females at this high frequency. In males, the negative response was concentrated on the lateral SC (lSC), whereas in females, the positive response was concentrated on the medial SC (mSC).

The temporal shape of the response to the 25‐Hz stimulus in the SC shed some light on the disparity in BOLD activation maps between males and females at 14 T (Figure 2J,K). Indeed, the shape was characterized by two positive peaks upon task onset and offset, with a lower signal plateau in‐between. In males, the response in the lSC plateaued below the baseline, which explains the negative *z* score in this region, whereas the signal plateau was above baseline in females, which translated into a positive *z* score.

In addition, we observed a marked difference in the shape of the SC response at 25 Hz between the two field strengths (Figure 2P,Q). At 9.4 T, the response did not display a double peak pattern but instead was more typical of a block response, with a large initial signal increase, followed by a slower continuous increase in amplitude during the stimulation, and a steep return to baseline upon task offset.

In V1, the response was positive at 1 Hz and negative at 25 Hz (as mentioned above) but was otherwise similar in shape and only varied in amplitude between males and females (Figure 2L) and across field strengths (Figure 2R).

Activation maps obtained from b200 time series showed positive BOLD response in the same regions as in GRE BOLD (Supplementary Figure S3), although covering a reduced area. Negative BOLD response was found at 25 Hz in the visual cortex but not in the SC and also covered a smaller area than in GRE‐BOLD.

### 3.2 ADC‐fMRI Is Still Sensitive to Vascular Response Following Visual Stimulation at 14 T

We compared activation maps obtained from BOLD and ADC‐fMRI at 14 T in females. We used an FIR function followed by unsupervised clustering to identify and isolate the voxels showing a significant signal variation during stimulation with low prior on the shape of the response function.

**FIGURE 3:**
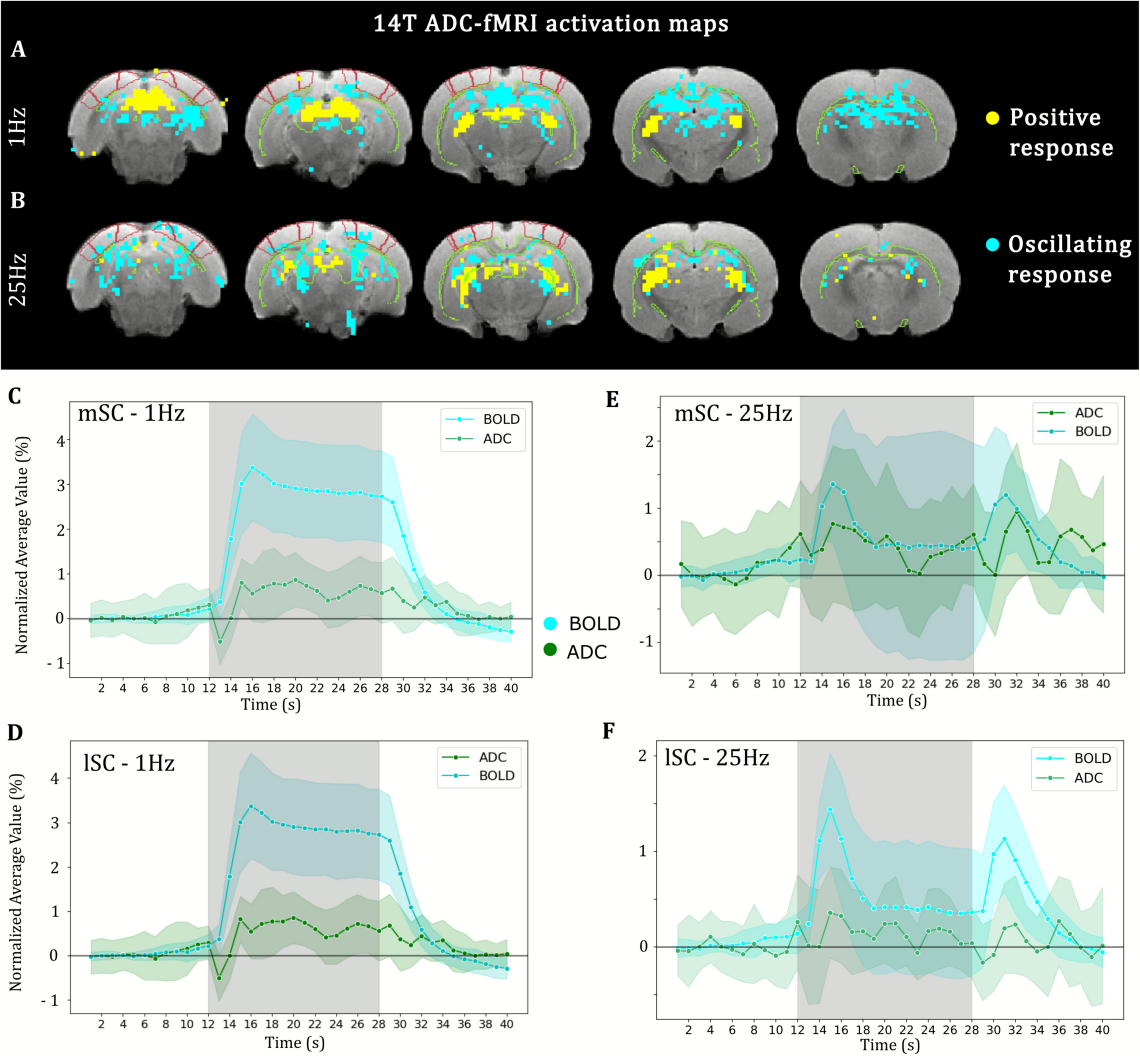
ADC response to 1‐ and 25‐Hz visual stimulation in comparison to BOLD at 14 T. (A, B) K‐means clustered voxels with average response to (A) 1‐Hz and (B) 25‐Hz visual stimulation. (C–F) Average response in the SC to (C, D) 1‐Hz and (E, F) 25‐Hz visual stimulation in (C and E) voxels in the medial SC, (D and F) voxels in the lateral SC. An oscillation was observed with frequency of about 0.25 Hz in the ADC response in the lateral SC at 25 Hz (F). This oscillation was not considered as relevant to the task as it was compatible with aliasing artifact from respiratory frequency. *n* = 6 females. Shading denotes standard deviation. Gray area represents stimulus duration.

We identified significant voxels with positive, negative, or an alternation of positive and negative response. In response to 1‐Hz visual stimulation, a large cluster of positive ADC response was found in the entire SC and in the DLGn, whereas negative ADC response was observed in the hippocampus and in the RSC (Figure 3A). In response to 25‐Hz visual stimulation, positive ADC response was found in the DLGn. The mSC and lSC had mixed positive and negative responses (Figure 3B). No significant response was found in V1 with either of the stimulation frequencies.

When plotting the temporal responses in the significant voxels, it appeared that the ADC response largely reflected the BOLD response (Figure 3C–E). Indeed, a positive ADC response to 1‐Hz visual stimulation was observed in both the medial and lateral parts of the SC (Figure 3C,D). The same voxels exhibited a strong positive BOLD response, which in addition displayed a very similar shape to the ADC response. Furthermore, the ADC response to 25‐Hz visual stimulation was also similar to BOLD in the mSC and lSC (Figure 3E,F).

### 3.3 ADC‐fMRI Detects Specific Signatures of Visual Stimulation in Both Subcortical Gray Matter and White Matter at 9.4 T

We investigated the ADC‐fMRI activation patterns in response to a visual stimulus independently from the BOLD effect at 9.4 T for both stimulation frequencies.

**FIGURE 4:**
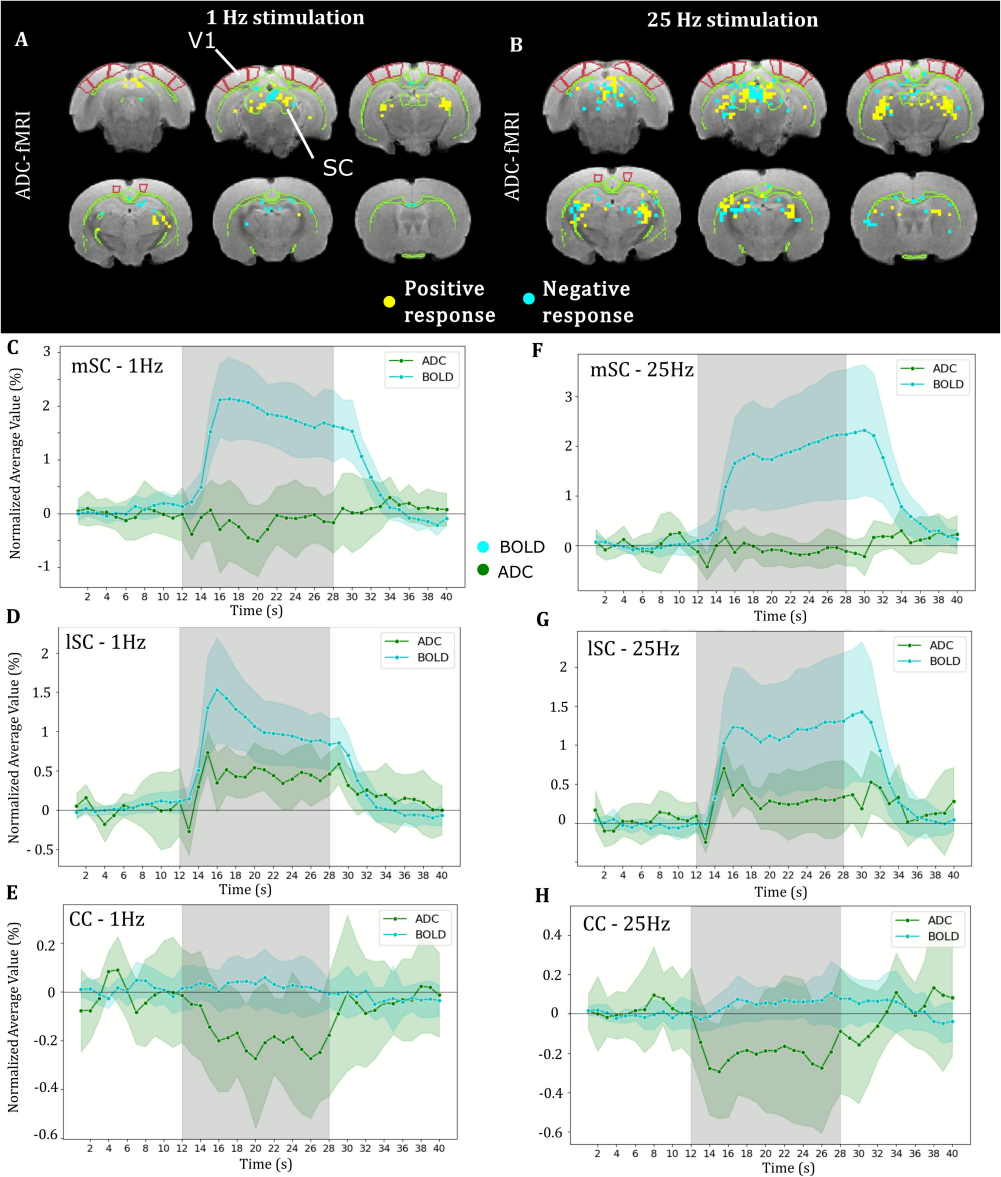
ADC response to 1‐ and 25‐Hz visual stimulation in comparison to BOLD at 9.4 T. (A, B) K‐means clustered voxels with average response to (A) 1‐Hz and (B) 25‐Hz visual stimulation. (C–E and F–H) Average response to (C–E) 1‐Hz and (F–H) 25‐Hz visual stimulation in (C and F) voxels with negative ADC response detected in the medial SC, (D and G) voxels with positive ADC response detected in the lateral SC, (E and H) voxels with negative ADC response detected in the corpus callosum (CC). *n* = 6 females. Shades represent standard deviation. Gray area represents stimulus duration.

In response to the 1‐Hz visual stimulus, clusters with negative ADC response (a signature of excitatory activity) were found in the medial part of the SC, the RSC, and the CC, whereas positive ADC response was found in the lateral part of the SC and in the DLGn (Figure 4A). No significant response was found in V1. Comparing ADC and BOLD average response in these group‐level clusters, we found negative ADC‐fMRI in the mSC in the presence of positive BOLD response (Figure 4C). The maximum amplitude of the ADC response was −0.5%, whereas the maximum amplitude of the BOLD response in the same area was 2.2%. Similar clusters were also found in the hippocampus. In the lateral part of the SC and in the DLGn, the positive BOLD response (+1.5% maximum amplitude) was accompanied by a positive ADC response with a very similar shape to BOLD and a maximum amplitude of +0.7%, suggesting residual vascular contributions to ADC time courses in these particular regions (Figure 4D). Remarkably, a negative ADC response (−0.3% maximum amplitude) was also detected in some clusters in the CC (Figure 4E), where no BOLD response could be observed. ADC responses are shown without corresponding BOLD response in Supplementary Figure S4.

Interestingly, ADC‐fMRI yielded very similar spatial patterns between the 25‐ and 1‐Hz visual stimulation (Figure 4B). Clusters with negative ADC response to 25‐Hz stimulation were found in the medial part of the SC and in the hippocampus. These voxels covered a larger area as compared to the 1‐Hz stimulus but showed lower amplitude (Figure 4F, −0.2%, vs. −0.4% at 1 Hz). In the same voxels, BOLD‐fMRI showed a positive response (+2.2% maximum amplitude). Similarly to the 1‐Hz stimulus, the average ADC response at 25 Hz in the lateral part of the SC was positive and showed a very similar shape to the BOLD response, with a maximum amplitude of +0.6% and +1.2%, respectively (Figure 4G). Finally, negative ADC response was also found in clusters in the CC with a maximum amplitude of −0.2% in the absence of any BOLD response (Figure 4H). No significant response was found in V1.

The activation patterns highlighted by ADC‐fMRI at 9.4 T were unique to this functional contrast. Indeed, an identical analysis pipeline of BOLD‐fMRI at 9.4 T using FIR yielded similar maps to the boxcar GLM, with a broader extent of activation than in ADC‐fMRI (Supplementary Figure S5).

## 4 Discussion

### 4.1 Results Highlight the Confounding Effect of the Vascular Origin of the BOLD Signal

As previously observed [8, 27], the increase in stimulation frequency induced a transition from positive to negative BOLD in the visual cortex. However, in our study, the BOLD response to 25‐Hz visual stimulation exhibited greater complexity than previously reported [8], especially in the SC, with variable responses across sexes and field strengths.

Our results showed for the first time that the response in the SC differed between males and females, at 14 T. Whereas the BOLD response was still positive throughout the SC in females, a negative BOLD response was visible only in the lateral part of the SC in males at 25 Hz. We found this difference to be mainly due to variations in the amplitude of the plateau between the two positive BOLD peaks that systematically occurred right after onset and end of the stimulus and solely at this frequency. These peaks were already described with a visual task [27] and an auditory task [42] and interpreted as “novelty” peaks. Cerebral arteries show larger inner diameter in male Sprague–Dawley rats than in females [43], whereas cerebral blood volume and vascular density are higher in females [44]. These differences in vasculature could lead to distinct BOLD responses between males and females, which can be a confounding factor for the interpretation of BOLD activation in terms of neuronal firing.

Interestingly, compared to Gil et al. [8], and despite using a similar experimental paradigm and stimulation frequencies, we observed a residual positive BOLD response in the SC at 25 Hz. This difference suggests that Sprague–Dawley rats present a higher FFF threshold than Long–Evans rats, possibly due to their albino condition. However, it could also be attributed to the younger average age of the rats in our study or to the known differences in vasculature between strains [45].

Our results revealed a consistent negative BOLD response in the rat auditory cortex at 1‐Hz stimulation, observed across both sexes and field strengths. Because the visual stimulus was not accompanied by any synchronized sound, it is unlikely that this response reflects a direct auditory response. Instead, the negative BOLD signal most plausibly arises from cross‐sensory interactions between visual and auditory systems, a phenomenon well documented in the human brain [46, 47].

Relative contributions from the microvascular, macrovascular, and extravascular compartments to the BOLD signal are highly dependent on the *B*_0_ field strength [48]. Extravascular contribution to the BOLD signal is dominant at ultrahigh field due to an increased extent of field distortions perpendicular to large radius vessels [10, 49]. Thus, discrepancies between responses at 9.4 and 14 T (evaluated in females only) may reflect specific involvements from areas close to large vessels. Using monocrystalline iron oxide nanoparticles injection for MR angiography of the rat brain, Lau et al. (2011) showed that the DLGn and lSC are irrigated by larger vessels than the mSC [50]. This regional difference in vascularization could lead to an increased contribution to the BOLD signal from the extravascular compartment in the lSC at 14 T vs. 9.4 T, which may explain the different response shapes across field strengths in this area. Interestingly, dw‐SE‐EPI with a *b*‐value of 200 s/mm^2^, mostly sensitive to extravascular dynamic dephasing [12], yields activation maps that are similar to those obtained with GRE‐EPI. This further supports the hypothesis that the BOLD contrast is dominated by extravascular contributions around small vessels. However, the broader spatial extent of significant activation in GRE‐EPI suggests that another source of contrast is also contributing with this sequence. Moreover, the larger discrepancy between dw‐SE‐EPI and GRE‐EPI for negative than for positive BOLD responses indicates that the relative weight of the underlying mechanisms may differ between positive and negative BOLD. As recent papers have pointed toward the important role of inhibitory neurons activity to explain negative BOLD signal [13, 14], distinct response in the medial and lateral subparts of the SC could also be partly attributed to different population of excitatory versus inhibitory neurons. These two subregions show different organizations and integrate separately to specific subnetworks [51, 52]. The medial part of the SC receives most of the input from V1 as part of the visual integration subnetwork, whereas the lSC is more integrated in visuomotor, sensorimotor, and escape‐approach subnetworks.

Our results suggest that the negative BOLD response in the lSC can thus be an effect of a higher proportion of inhibitory neurons, of the proximity of large veins amplifying the BOLD effect, or of a combination of both.

### 4.2 ADC‐fMRI Detects Neuronal Activation With High Spatial Specificity and Sensitivity to White Matter at 9.4 T

ADC‐fMRI acquired at 14 T showed a response similar to BOLD, suggesting vascular contribution at this field strength despite our acquisition scheme mitigating the main sources of BOLD contamination to the ADC time courses: T_2_ weighting (by taking the ratio of two successive diffusion‐weighted images to calculate an ADC), direct blood water contributions (by using *b*‐values ≥ 200 s/mm^2^), and cross‐terms between background susceptibility gradients and diffusion gradients (by using cross‐term compensated gradient waveforms). This could be due to remaining IVIM effects at the lowest *b*‐value [23]. Unsurprisingly, this vascular contribution was weaker at a lower field strength, as ADC‐fMRI responses at 9.4 T did not mirror BOLD responses in some brain areas.

In what follows, we discuss ADC‐fMRI responses in females at 9.4 T exclusively. A negative ADC response was found in the mSC at both visual stimulation frequencies, but with different amplitudes (−0.5% at 1 Hz vs. −0.2% at 25 Hz). An ADC decrease has been previously associated with excitatory neuronal activity and reported even in the absence of vasculature [26, 53, 54, 55, 56]. Excitatory activity in the mSC at both frequencies is corroborated by the strong positive BOLD response observed in the same brain area at this field strength.

A negative ADC response was also found in the hippocampus. The hippocampus, although not considered part of the visual pathway per se, is involved in high‐order visual processes [57]. In particular, in the rodent brain, the hippocampus is connected to the medial part of the SC as part of the head direction network [51]. Whereas these hippocampal voxels were packed within the large area of positive response in the SC using BOLD‐fMRI, in ADC‐fMRI they constituted a distinct cluster. This result highlights the potential of ADC‐fMRI to detect activation with improved spatial specificity [25]. Of note, a previous study of rat forepaw stimulation reported rather an increase in ADC in the subcortical regions involved, directly or indirectly, in the somatosensory pathway—thalamus, hippocampus, striatum—in the absence of a detectable BOLD response in those areas [58]. When not overwhelmed by vascular contamination, the polarity of the ADC response may yield an accurate indication of tunable excitatory/inhibitory balance in subcortical regions participating in a variety of sensory and higher‐order function pathways [54, 55]. In our current data, unfortunately, positive ADC responses can only be interpreted as resulting from vascular contamination, due to their temporal and spatial similarity to positive BOLD response (see Limitations and Perspectives).

A negative ADC response was also detected in a few clusters located in white matter and more specifically in the CC and hippocampal commissure, suggesting neuronal firing of interhemispheric projections, which may be linked to bilateral stimulation. In comparison, no specific activation of white matter was detected using BOLD‐fMRI using either boxcar or FIR response functions. This is consistent with similar recent findings of specific ADC‐fMRI detection of activation in the human optic radiation [26]. Although the association between the detected white matter bundles and the task needs more investigation, this result reinforces previous findings from both task and resting‐state fMRI [24, 25], highlighting the improved sensitivity of ADC‐fMRI to white matter neural activity.

### 4.3 Limitations and Perspectives

No significant ADC response was found in V1, whereas this activation was expected and measured with BOLD. The main explanation is likely the low amplitude of response of this region to the task. Indeed, BOLD response in V1 showed an amplitude reduced by a factor of 10 compared to the amplitude of the response in the mSC. As the amplitude of the ADC response is lower than BOLD, in V1 it probably stays under detectability threshold. The weak BOLD response observed in V1 can be attributed to a combination of factors: first, a relatively low SNR in this region and, second, our visual stimulation setup. Compared to Gil et al. [8], we positioned the optic fibers parallel to the head of the rat and did not use a diffuser, relying only on the reflection of the light on the inner walls of the bore for stimulation.

In addition to the negative ADC clusters, positive ADC clusters were found in the lSC and in the DLGn at 9.4 T. The shape of the ADC response in those areas was very similar to the BOLD response for both stimulation frequencies. Such similarity strongly implies a BOLD contribution to the ADC time series, despite our efforts to minimize them. Interestingly, a negative ADC response was nonetheless found in the mSC, where the positive BOLD response was the strongest. As discussed above, the vascularization of the lateral and medial parts of the SC is very different. This suggests that vascular contribution to ADC response highly depends on the proximity to large vessels, resulting in more contamination in the lSC than the mSC. Furthermore, at 14 T, where the magnetic susceptibility effects are even more pronounced, the ADC response maps largely mirrored BOLD maps, suggesting more widespread BOLD contamination.

Reducing BOLD contribution is crucial to fully leverage the higher specificity of ADC‐fMRI. In this study, the BOLD contribution to ADC was minimized using cross‐term compensated waveforms, calculating an ADC from interleaved *b*‐values instead of single *b*‐value diffusion‐weighted (and T_2_‐w) time courses, and employing a minimal *b*‐value of 200 s/mm^2^ to reduce direct signal from the intravascular compartment. However, several measures may still be taken for further minimizing the BOLD contribution. First, the acquisition of alternating *b*‐values leads to imperfect T_2_‐weighting cancellation in the computation of the ADC, as the T_2_ may vary between two successive images used to compute one ADC volume. Further studies are needed to cancel more thoroughly any T_2_ contribution, e.g., by modifying the acquisition scheme to acquire both *b*‐values within the same TR [59]. Another strategy could be to use ultrafast imaging (i.e., with very short TR) for increased temporal resolution and reduced T_2_ variation between successive images [55, 60]. However, very short TRs typically limit the acquisition to single‐slice [60] or line scanning [55] and substantially reduce SNR because the longitudinal magnetization has insufficient time to recover between excitations. In addition, such protocols typically incur high SAR, which further constrains their practical use. Second, as already mentioned, as the amplitude of the BOLD signal heavily relies on field strength, it is expected that reducing the field strength will lead to a reduction of this contribution. Indeed, we observed higher BOLD contributions to ADC‐fMRI at 14 T than at 9.4 T. In comparison, previous literature studies on 3 T ADC‐fMRI were relatively insensitive to BOLD [24, 25, 61 ].

Although the precise cellular origin of the ADC‐fMRI contrast remains to be firmly established, growing evidence suggests it is not of vascular origin. Indeed, previous numerical simulations have shown that realistic neural cell swelling can induce a detectable decrease in ADC due to increased extracellular tortuosity and shift of relative weights toward a more restricted intracellular compartment [26]. Recent work using a hypercapnia challenge that also induces vasodilation without neural activity failed to detect an ADC response at 3 T [61]. Furthermore, in the current study, as in other previous works [26], the strongest ADC‐fMRI response was detected in regions with reduced vascularization. In future work, using higher *b*‐values would be interesting to isolate more specifically cellular contribution [62, 63]; however, this would remain a challenge as increasing *b*‐values would lead to heavily reduced SNR.

To conclude, in this study, we compared the BOLD and ADC‐fMRI responses to visual stimuli in the rat using different frequencies and field strengths. Our results highlighted the potential confounding effect of vasculature on BOLD contrast, especially across field strengths and sexes. In comparison, ADC‐fMRI enabled the identification of distinct activation clusters in the mSC, hippocampus, and CC, not systematically matched by the BOLD contrast. However, more methodological development is needed to further reduce BOLD contribution to ADC‐fMRI at ultrahigh field in highly vascularized brain areas, in order to fully exploit the sensitivity and specificity of ADC‐fMRI under optimal conditions. Overall, this study highlights the complementarity of ADC‐fMRI to BOLD. Although the sensitivity of BOLD‐fMRI is unmatched and led to a better understanding of the FFF mechanism, the involvement of inhibitory neurons is largely convolved and confounded by local vascularization. As a comparison, ADC‐fMRI holds the potential of a very specific contrast to measure excitation or suppression of neuronal activity.

## Author Contributions

**Jean‐Baptiste Pérot:** conceptualization, data curation, formal analysis, investigation, methodology, visualization, writing – original draft. **Andreea Hertanu:** data curation, methodology, investigation, writing – review and editing. **Arthur Spencer:** methodology, writing – review and editing. **Jasmine Nguyen‐Duc:** methodology, writing – review and editing. **Nikolaos Molochidis:** methodology, writing – review and editing. **Valerio Zerbi:** methodology, writing – review and editing. **Maxime Yon:** methodology, writing – review and editing. **Ileana Jelescu:** conceptualization, funding acquisition, project administration, resources, supervision, writing – original draft.

## Funding

This work was supported by ERC Starting Grant “FIREPATH,” Staatssekretariat für Bildung, Forschung und Innovation No. MB22.00032. I.J. is supported by Swiss National Science Foundation (SNSF) Eccellenza Fellowship No. 194260. V.Z. and N.M are supported by SNSF Eccellenza PCEFP3_203005.

## Conflicts of Interest

The authors declare no conflicts of interest.

## Supporting information


**Figure S1:** Image processing pipeline. Representative images from a single subject of GRE‐EPI time series and dw‐SE‐EPI time series with a *b*‐value of 200 or 1000 s/mm^2^ are shown at principal steps of preprocessing. While similar pipeline was performed, it is shown for both 14 and 9.4 T for comparison. Multivariate template was the same for both field strengths and consisted in T_2_‐w, GRE‐EPI, and SE‐EPI (*b* = 0 s/mm^2^) images. Temporal SNR from a spherical ROI in the cortex is shown for each time series at the end of the preprocessing pipeline, before registration, as well as in the segmented visual cortex (VC) and superior colliculus (SC).
**Figure S2:** Segmentation of the medial and lateral parts of the superior colliculus (SC).
**Figure S3:** Activation maps of dw‐SE‐EPI with a *b*‐value of 200 s/mm^2^ in the brain of male and female rats at 14 and 9.4 T. (A–F) Cluster‐corrected group‐level activation maps of significant positive (red–yellow) and negative (blue–light blue) response to 1‐Hz (A–C) and 25‐Hz (D–F) visual stimulation in (A and D) males acquired at 14 T (*n* = 6, |*Z*| > 1.5, |*Z*|_max_ = 3.9), (B and E) females acquired at 14 T (*n* = 6, |*Z*| > 1.5, |*Z*|_max_ = 6.1), (C and F) females acquired at 9.4 T (*n* = 6, |*Z*| > 1.5, |*Z*|_max_ = 13.9.
**Figure S4:** ADC responses to visual stimulation in females at 9.4 T. This figure presents the same responses as Figure 4 without BOLD response for better visualization. Average response to (A–C) 1‐Hz and (D–F) 25‐Hz visual stimulation in (A and D) voxels with negative ADC response detected in the medial SC, (B and E) voxels with positive ADC response detected in the lateral SC, (C and F) voxels with negative ADC response detected in the corpus callosum (CC). *n* = 6 females. Shades represent standard deviation. Gray area represents stimulus duration.
**Figure S5:** Activation maps of BOLD‐fMRI resulting from the GLM analysis using an FIR response function at 9.4 T in female rats, in response to (A) 1‐Hz and (B) 25‐Hz visual stimulation, mirroring the ADC‐fMRI FIR analysis (Figure 4A,B). Similar information to GLM with a boxcar response function is found (Figure 2). No specific activation in white matter regions can be observed.

**Data S1:** Supporting Information

## Data Availability

The data, code, protocols, and key laboratory materials used and generated in this study are listed in a Key Resource Table alongside their persistent identifiers at https://doi.org/10.5281/zenodo.15705581.
