# Supplementary material for: Complementarity of BOLD and ADC‐fMRI in Mapping Brain Visual Processing in the Rat
Source: NMR Biomed. 2026 Feb 3;39(3):e70231. doi: 10.1002/nbm.70231 (PMC12865745; doi:10.1002/nbm.70231)
Supplement: Supplementary file 2 — Data S1: Supporting Information [file NBM-39-e70231-s002.docx]

# Supplementary Figures


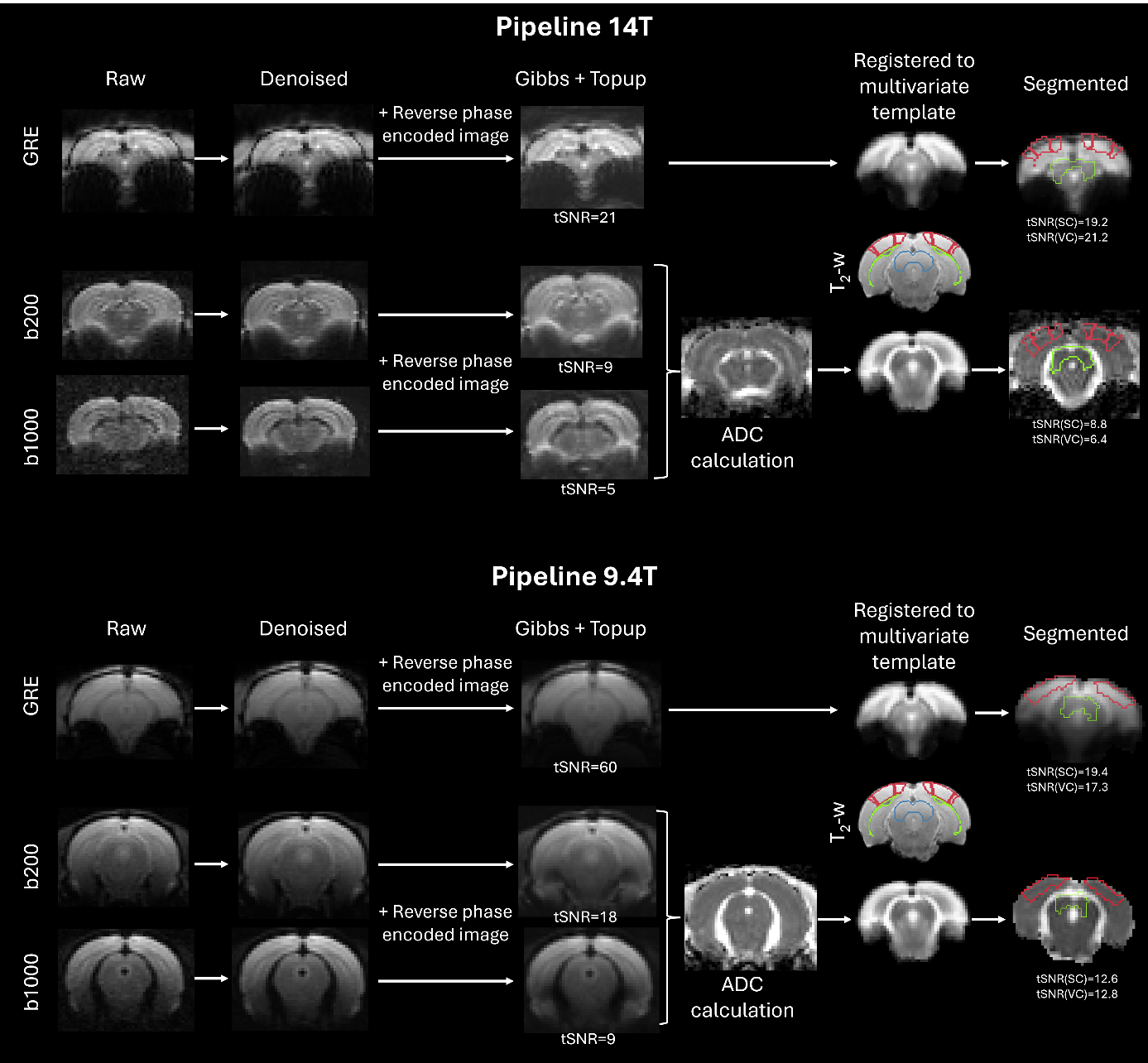


Supplementary Figure 1: Image processing pipeline. Representative images from a single subject of GRE-EPI time series and dw-SE-EPI time series with a b-value of 200 s/mm² or 1000 s/mm² are shown at principal steps of preprocessing. While similar pipeline was performed, it is shown for both 14T and 9.4T for comparison. Multivariate template was the same for both field strengths and consisted in T_2_-w, GRE-EPI, and SE-EPI (b=0 s/mm²) images. Temporal SNR from a spherical ROI in the cortex is shown for each time series at the end of the preprocessing pipeline, before registration, as well as in the segmented visual cortex (VC) and superior colliculus (SC).


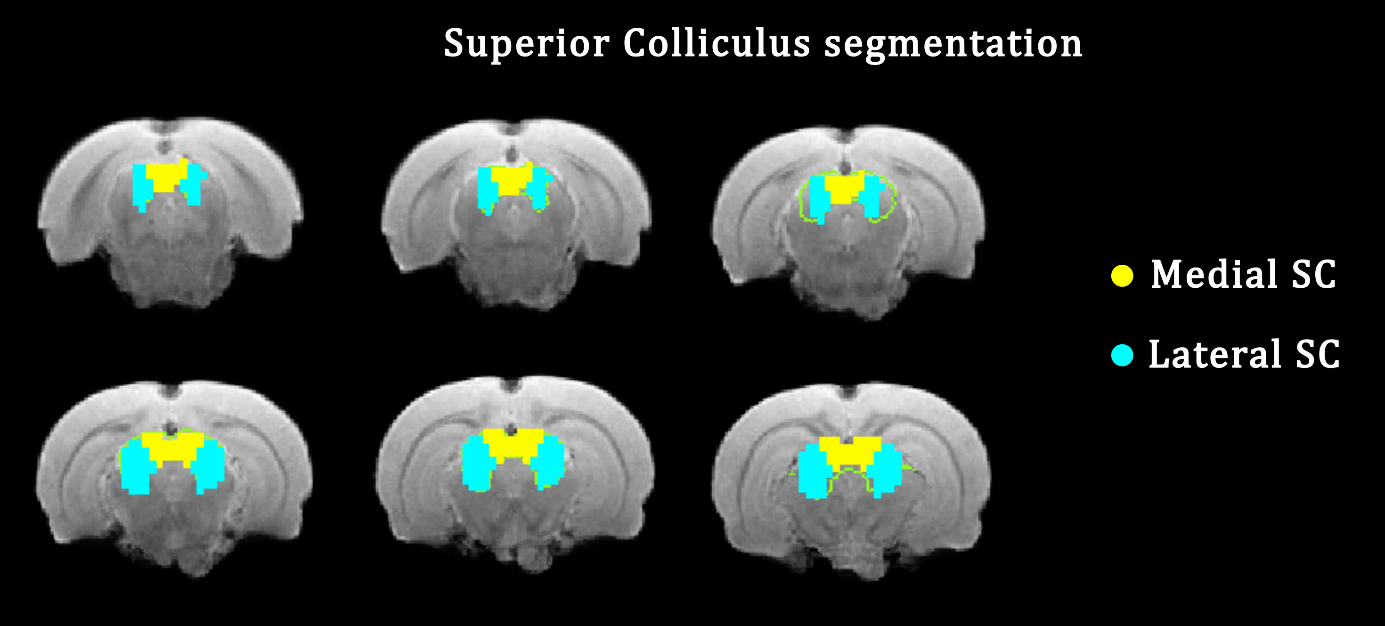


**Supplementary Figure 2: Segmentation of the medial and lateral parts of the superior colliculus (SC)**

**
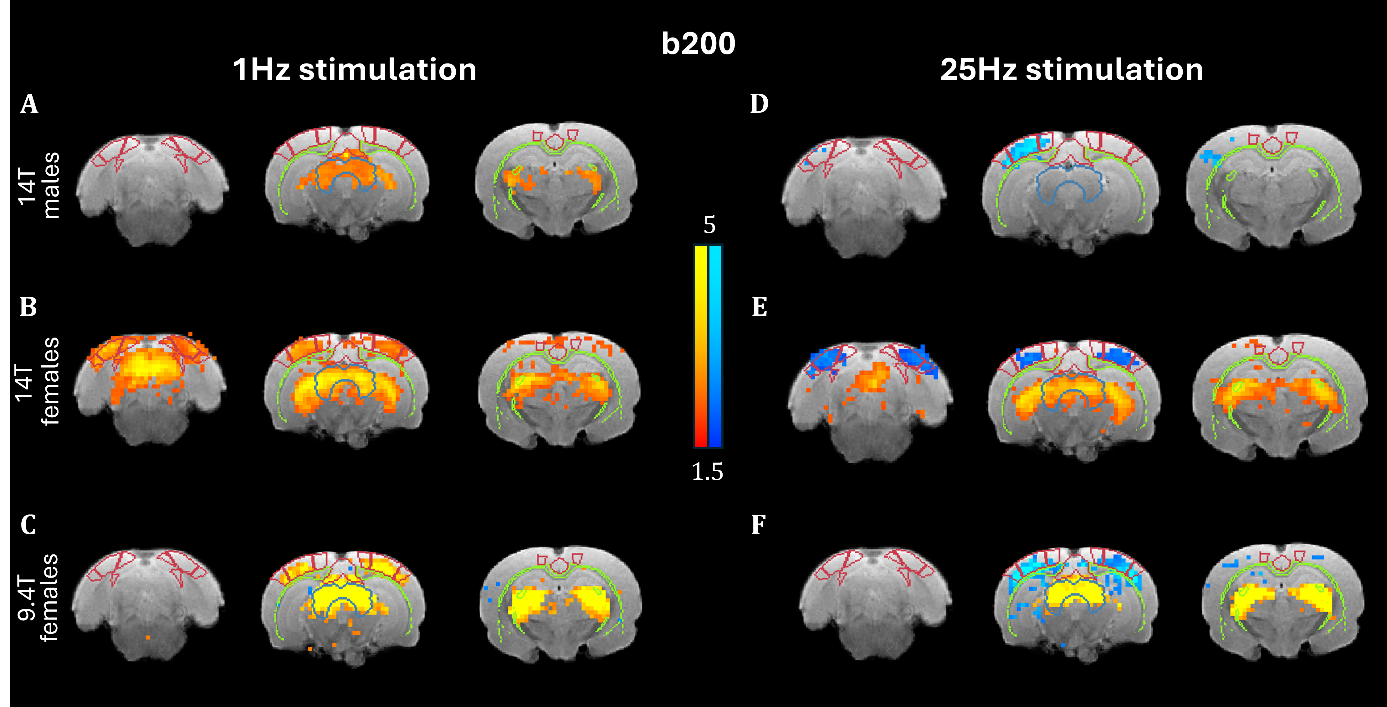
**

**Supplementary Figure 3: Activation maps of dw-SE-EPI with a b-value of 200 s/mm² in the brain of male and female rats at 14T and 9.4T.** A-F: Cluster-corrected group-level activation maps of significant positive (red-yellow) and negative (blue-light blue) response to 1Hz (A-C) and 25Hz (D-F) visual stimulation in (A & D) males acquired at 14T (n=6, |Z|>1.5, |Z|_max_ = 3.9), (B & E) females acquired at 14T (n=6, |Z|>1.5, |Z|_max_ = 6.1), (C & F) females acquired at 9.4T (n=6, |Z|>1.5, |Z|_max_ = 13.9.

**
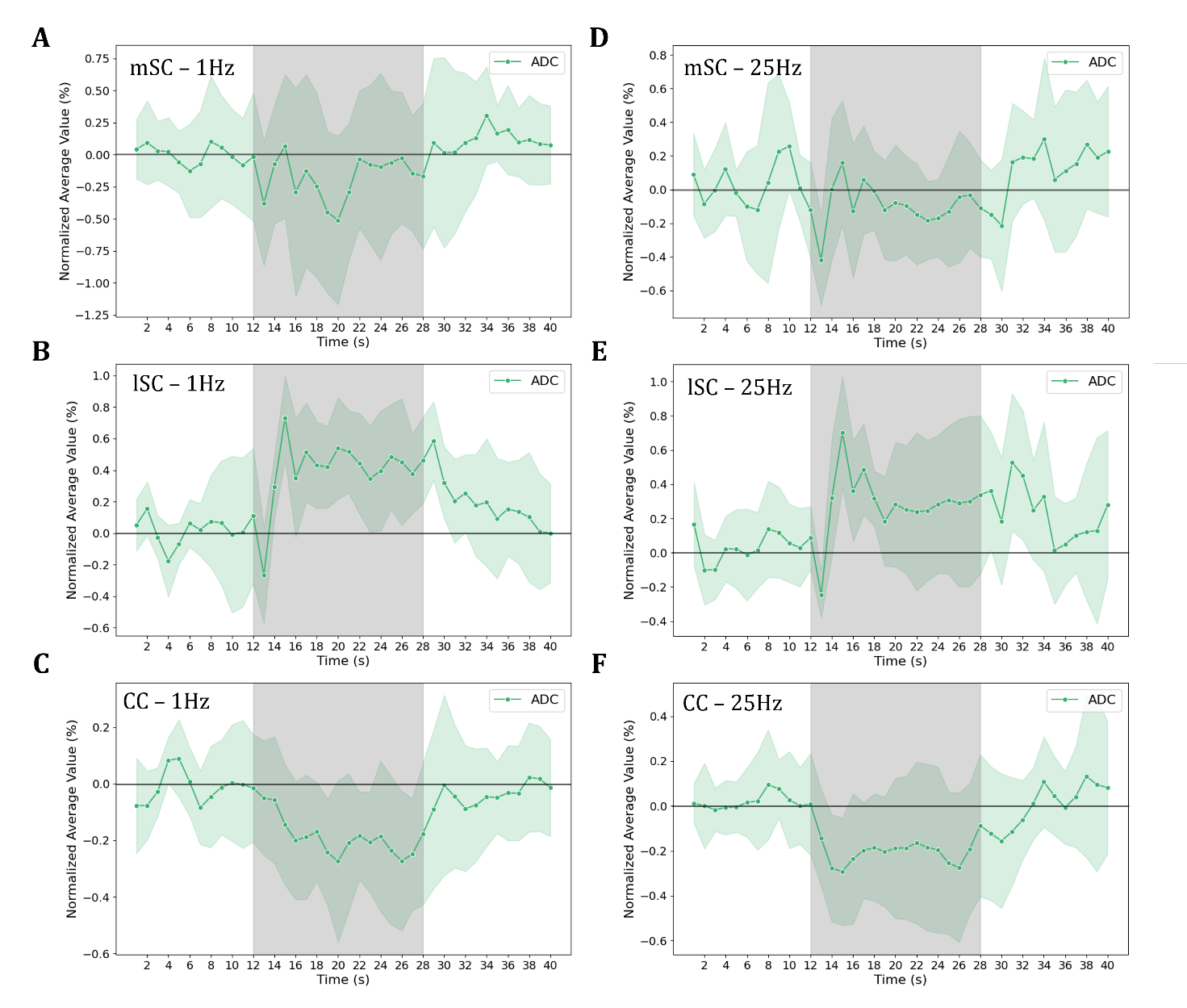
**

**Supplementary Figure 4: ADC responses to visual stimulation in females at 9.4T.** This figure presents the same responses as Figure 4 without BOLD response for better visualization. Average response to (A-C) 1Hz and (D-F) 25Hz visual stimulation in (A & D) voxels with negative ADC response detected in the medial SC, (B & E) voxels with positive ADC response detected in the lateral SC, (C & F) voxels with negative ADC response detected in the corpus callosum (CC). N=6 females. Shades represent standard deviation. Grey area represents stimulus duration.


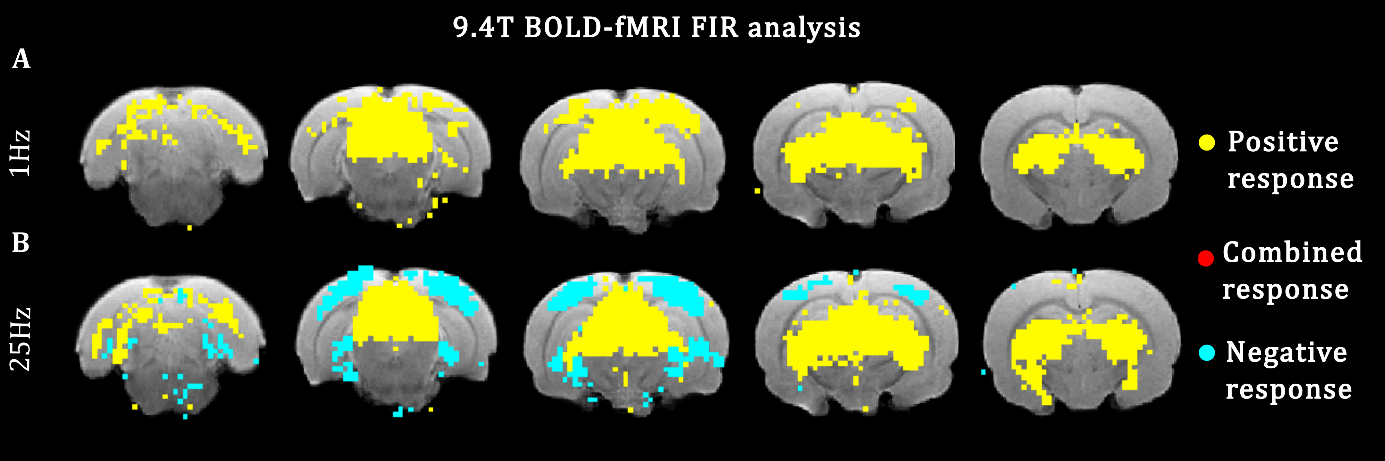


**Supplementary Figure 5: Activation maps of BOLD-fMRI resulting from the GLM analysis using a FIR response function** at 9.4T in female rats, in response to (A) 1Hz and (B) 25Hz visual stimulation, mirroring the ADC-fMRI FIR analysis (Figure 4A-B). Similar information to GLM with a boxcar response function is found (Figure 2). No specific activation in white matter regions can be observed.
